# In-silico evaluation of an artificial pancreas achieving automatic glycemic control in patients with type 1 diabetes

**DOI:** 10.3389/fendo.2023.1115436

**Published:** 2023-01-30

**Authors:** Wenping Liu, Ting Chen, Bingjin Liang, Yanran Wang, Haoyu Jin

**Affiliations:** Institute of Medical Devices, Guangdong Food and Drug Vocational College, Guangzhou, China

**Keywords:** automated artificial pancreas, general predictive control, adaptive control weighting parameter, hypoglycemia prevention, effective and safe glycemic control

## Abstract

Artificial pancreas (AP) is a useful tool for maintaining the blood glucose (BG) of patients with type 1 diabetes (T1D) within the euglycemic range. An intelligent controller has been developed based on general predictive control (GPC) for AP. This controller exhibits good performance with the UVA/Padova T1D mellitus simulator approved by the US Food and Drug Administration. In this work, the GPC controller was further evaluated under strict conditions, including a pump with noise and error, a CGM sensor with noise and error, a high carbohydrate intake, and a large population of 100 in-silico subjects. Test results showed that the subjects are in high risk for hypoglycemia. Thus, an insulin on board (IOB) calculator, as well as an adaptive control weighting parameter (AW) strategy, was introduced. The percentage of time spent in euglycemic range of the in-silico subjects was 86.0% ± 5.8%, and the patient group had a low risk of hypoglycemia with the GPC+IOB+AW controller. Moreover, the proposed AW strategy is more effective in hypoglycemia prevention and does not require any personalized data compared with the IOB calculator. Thus, the proposed controller realized an automatic control of the BG of patients with T1D without meal announcements and complex user interaction.

## 1 Introduction

Type 1 diabetes (T1D) is an autoimmune disease characterized by chronic hyperglycemia. In patients with T1D, immune-mediated destruction of the pancreatic β cells occurs, and the pancreases produce very little or no insulin (1). In 2021, over 1.2 million children and adolescents had T1D mellitus (T1DM), and this number is increasing annually (2, 3). By 2030, 578 million people are predicted to suffer diabetes (including types 1 and 2), and this number will increase to 700 million by 2045 (4).

**Figure 1:**
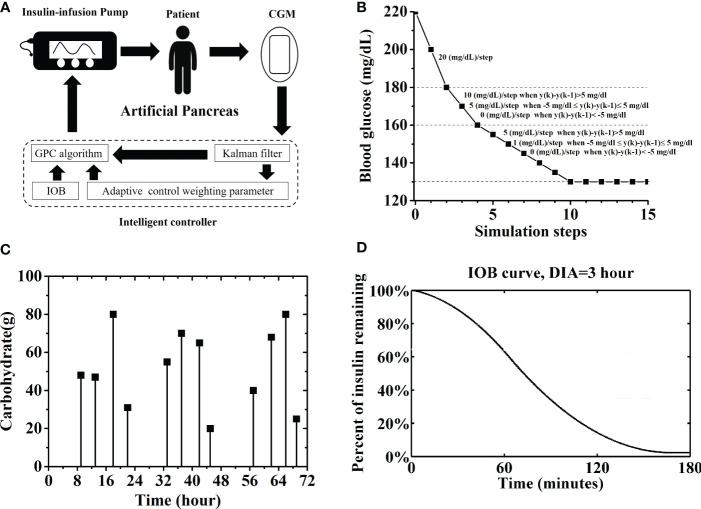
**(A)** Sketch map of an AP system, including a CGM, an insulin pump and an intelligent controller. Here, the intelligent controller was based on general predictive control (GPC) algorithm. A Kalman man filter, an IOB calculator and an AW strategy were introduced into the controller. **(B)** Adaptive reference glucose trajectory. **(C)** Carbohydrate and time of twelve unannounced meals. **(D)** IOB curve for prediction the active insulin amount, DIA is set as 3 hours.

Although the causes of T1D are not fully understood, patients with T1D can live healthy lives with appropriate daily insulin injections. Artificial pancreas (AP) is also a useful tool for maintaining the blood glucose (BG) of T1D patients within the euglycemic range (70–180 mg/dL) (5). It comprises a continuous glucose monitoring (CGM), an insulin pump, and an intelligent controller that connects these two devices (**Figure 1A**). Studies revealed that patients with T1D who used CGM and multiple insulin injections had lower hemoglobin A_1c_ levels than those receiving usual care (6). The use of AP system improves glycemic control and reduces the risk of hypoglycemia in different age groups with T1D compared with conventional or sensor-augmented pump therapy (7). The world’s first AP system, the Medtronic’s MiniMed 670G system, was approved by the US Food and Drug Administration (FDA) in 2016 and has been commercially available (8). This system not only reduces the patient workload and achieves good glucose control but also reduces the threat of diabetes-related complications (9). Three other AP systems, including Tandem Control-IQ in the US and Diabeloop and CamAPS FX in Europe, have received regulatory approval since then. Several AP systems are also in development or under clinical trials. The international diabetes closed-loop trial evaluated a mobile AP application that runs on Android smartphones and use Bluetooth to wirelessly communicate with the CGM and insulin pump. It achieved certain levels of reliability and wireless connection stability (10). Melissa J. Schoelwer et al. proposed a slim X2 Control-IQ hybrid closed-loop system using parameters that were based on total daily insulin, which was tested in 20 participants and proved to be effective and safe (11). Nowadays, three do-it-yourself AP systems, including OpenAPS, AndroidAPS, and Loop, are available on websites (12). Individuals can also build their own AP systems following the instructions and algorithms of those AP systems (13).

Despite the great achievement in the development of AP systems in recent years, the usability of these devices in the real-world setting is the main challenge. In other words, they are not entirely automated, thereby requiring user interaction to deliver mealtime insulin boluses (14). Compared with other control algorithms, such as proportional-integral-derivative (PID) control and model predictive control (MPC), generalized predictive control (GPC) algorithm is an adaptive control method and does not require knowledge on the initial parameters or precise glucose–insulin relationship. It could calculate the optimal insulin injection rate by minimizing the deviation of the predicted BG values from a reference glucose trajectory. The predicted BG values is calculated from an autoregressive integrated moving-average model with exogenous inputs (ARIMAX). GPC algorithm has been applied for AP system, and some improvements have been made to improve its performance. For example, Meriyan and Sato T et al. proposed a time-varying reference trajectory with fixed slopes for glucose concentration instead of a single set-point trajectory (15). Some design parameters of the GPC, such as the softening factor and forgetting factor, significantly affected the system output and should be established cautiously (15, 16). Turksoy et al. introduced some physiological signals, such as energy expenditure and galvanic skin responses, to the GPC model for post-exercise hypoglycemia prevention. Although these signals achieved a good performance, they increased the complexity of the system (17). Mirko Messori et al. introduced a novel kernel-based nonparametric approach and a constrained optimization to realize model individualization. However, model identification and validation relies on the collected patient data, and the constrained optimization further requires to postulate a model structure as prior knowledge (18). Dassau and Hyunjin Lee et al. developed several meal detection and meal size estimation algorithms for AP controller to alarm individuals or to deliver a bolus automatically (19, 20). However, it was constrained by the threshold values set for different individuals. Thus, an intelligent controller was proposed based on GPC for AP, which calculated the insulin injection rate by only regarding the BG concentration measured by the CGM without information on the dose and timing of carbohydrates intake (21). Two adaptive strategies, including an adaptive reference glucose trajectory and an adaptive softening factor, were proposed for the GPC controller to increase system robustness when patients have normal BG values, as well as the tracking speed when patients have high hyperglycemia risk. Tests with the UVA/Padova T1DM simulator (T1DMS) approved by the FDA showed that it effectively controlled the BG of in-silico subjects under normal conditions. Here, the performance of the GPC controller was further evaluated with strict conditions, including a pump with noise and error, a CGM sensor with noise and error, a high carbohydrate (CHO) intake, and a large population of 100 in-silico subjects. Test results showed that the subjects were prone to high risk of hypoglycemia. Thus, an insulin on board (IOB) calculator, as well as an adaptive control weighting parameter (AW) strategy was introduced (**Figure 1A**). The performance of the GPC controller was significantly improved with them. The percentage of time spent in the euglycemic range (TIR) of the in-silico subjects was 86.0% ± 5.8%, and the patient group had a low risk of hypoglycemia with the GPC+IOB+AW controller. Moreover, the proposed GPC controller is effective in hypoglycemia prevention without the need for personalized data, complex user interaction, and meal announcements, which realizes an automatic control of the BG of patients with T1D.

## Methods and materials

### GPC controller

A GPC controller proposed in our previous research is applied for BG regulation (21). The GPC controller only reads the BG sent from the CGM sensor, and the optimal insulin rate was calculated. A basic Kalman filter was added to smooth the BG readings (**Figure 1A**). The Kalman filter was built using the Simulink tool in the MATLAB software environment without any personalized data (Details are shown in Supplemental Appendix S1).

Insulin injection rate is computed by minimizing J in the following function.


$$\begin{array}{l} J=\sum_{j=1}^{n}{\left[y\left(k+j\right)-w\left(k+j\right)\right]}^{2}+\sum_{j=1}^{m}\lambda {\left[\Delta u\left(k+j-1\right)\right]}^{2}\;\\ w\left(k+j\right)=\alpha^{j}y\left(k\right)+\left(1-\alpha^{j}\right)y_{r}\;\;\left(j=1,2,\ldots ,n)(1\right) \end{array}$$

where *y*(*k* + *j*) represents the *j*-step-ahead prediction of the process output, and Δ*u* (*k + j−*1) denotes the incremental control input at the (*k + j−*1) sampling step. *n*, which denotes the output prediction horizon, is set as 8. m, which denotes the control horizon, is set as 4. *Λ*, which denotes the control weighting parameter, is set as 0.2. *y*(*k*) represents the current BG. *y_r_* denotes the adaptive reference glucose trajectory, which has slopes that are adjusted in accordance with variations in the BG measured in the past two steps (i.e., *y*(*k*) and *y*(*k−*1) (**Figure 1B**). α, which denotes the adaptive softening factor, is designed as $\text{α}=\tau^{-\left|y_{\left(k\right)}-y_{\left(k-1\right)}\right|},\;\tau =1+\frac{\left|y_{\left(k\right)}-\bar{y}\right|}{\bar{y}}$ , where $\bar{y\;}$ = 130 mg/dl (21).

The *j*-step-ahead prediction *y*(*k* + *j*) is estimated using an autoregressive integrated moving-average model with exogenous inputs (Function 2) and the Diophantine equation (Function 3).*u*(*k*) is the control input variable (insulin infusion rate), *ξ* (*k*) denotes the zero-mean white noise, and Δ = (1−*z* ^-1^) denotes the integration. The minimization of Function (1) provides the optimal control action (insulin infusion rate). The parameter design and the solution process are introduced in our previous research in detail (21).


$$A(z^{-1})y\left(k\right)=B\left(z^{-1}\right)u\left(k-1\right)+C\left(z^{-1}\right)\xi \left(k\right)/\Delta$$

$$A\left(z^{-1}\right)=1+a_{1}z^{-1}+\ldots +a_{na}z^{-na}$$

$$B\left(z^{-1}\right)=b_{0}+b_{1}z^{-1}+\ldots +b_{nb}z^{-nb}$$

(2) $$C\left(z^{-1}\right)=1+c_{1}z^{-1}+\ldots +c_{nc}z^{-nc}$$

$$1=E_{j}(z^{-1})A(z^{-1})\Delta +z^{-j}F_{j}(z^{-1})$$

$$E_{j}(z^{-1})=e_{j0}+e_{j1}z^{-1}+\ldots +e_{j,j-1}z^{-j+1}$$

(3) $$F_{j}(z^{-1})=f_{j0}+f_{j1}z^{-1}+\ldots +f_{jn}z^{-n}$$

### Software, scenario design, and data analysis

The GPC controllers were built and tested using the UVA/Padova T1DMS version 3.2.1, which embodies the biophysiological parameters of the FDA-accepted in silico populations (22). The software has two versions, namely, the academic and commercial versions. The former contains 10 adult subjects, whereas the latter contains 100 adult subjects. In the academic version, Subject 09 was excluded because the endogenous glucose production of this patient was suppressed even 6 h after meals, thereby leading to hypoglycemia (23).

In this work, the GPC controllers were first tested using 9 subjects and then sent to the Epsilon Group to test with 100 subjects. The parameter settings in the two versions of the simulator were identical, as stated below.

A CGM sensor with noise and error was used, as well as a pump with noise and error. Sensor noise and error are generated in the T1DMS with hand-written script (24). Pump noise and error are generated with two Gaussian-distributed random signal generators in the T1DMS, respectively. Details are shown in Supplemental Appendix S2. Totally, the sensor error (including noise) has a mean of 0.76 mg/dl and a standard deviation of 11 mg/dl. This sensor simulation model is believed to provide worst-case scenario sensor errors and the real sensor errors tend to be smaller during controlled inpatient clinical trials (25).

The same scenario used by Kamuran Turksoy et al. was reproduced in this study (26). All patients from the simulator were simulated over three days (72 hours), and the sampling time was set as 5 min. A total of 12 unannounced meals were provided and lasted 15 min each. **Figure 1C** shows the multiple meals provided during the testing period. The scenario was repeated 30 times for each in-silico subject. The BG trace of each in-silico subject was recorded. The average BG value was calculated for the patient group, as well as the percentages of time spent in the severe hypoglycemia range (BG ≤ 50 mg/dL), the hypoglycemia range (BG ≤ 70 mg/dL), the hyperglycemia range (BG > 180 mg/dL), and the severe hyperglycemia range (BG > 300 mg/dL) (27).

The percentage of time spent in euglycemic range (TIR) was used to evaluate the efficacy of the GPC controller. Two risk indexes provided by the UVA/Padova T1DMS, namely, low (LBGI) and high BG indexes (HBGI), were used to evaluate the safety of the GPC controller. LBGI refers to a measure of the frequency and extent of low BG readings, whereas HBGI refers to a measure of the frequency and extent of high BG readings. LBGI can be used to identify minimal- (LBGI< 1.1), low- (1.1 ≤ LBGI< 2.5), moderate- (2.5 ≤ LBGI< 5), and high-risk (LBGI > 5.0) of the patient subject for hypoglycemia. HBGI can be used to identify minimal- (HBGI< 5.0), low- (5.0 ≤ HBGI< 10.0), moderate- (10.0 ≤ HBGI< 15), and high-risk (HBGI > 15.0) of the patient subject for hyperglycemia.

The TIR, HBGI, and LBGI of the in-silico patient group was analyzed statistically to determine whether the GPC controller was significantly improved with the IOB or AW strategy (p ≤ 0.05 or p ≤ 0.01). The statistical analysis was performed in two steps: First, F-tests were performed to compare the variances of the BGC percentages of the two groups. Second, T-tests were conducted to compare their means. Unequal variance T-tests were applied when the variances were not equal.

### IOB calculator

The insulin will take a lag to reach the bloodstream and influence cell behavior after injection. The IOB refers to the percentage of active insulin units in a patient’s body. Several pump companies have considered the amount of IOB to avoid hypoglycemia and to keep the patient’s BG within the euglycemic range (28). IOB is a function of the duration of insulin activity (DIA) and the number of previous insulin amount (29). DIA is an individualized data that vary because of blood flow, injection site, temperature, and exercise (17, 30). Here, the IOB calculator was built by following the instruction of the OpenAPS on the website (31). The DIA is set as 3 h, and the IOB curve is shown in **Figure 1D**. Thus, the estimated IOB will be subtracted from *u*(*k*), and only a basal insulin infusion rate (around 60 p mol/min) will be used if *u*(*k*) is smaller than IOB.

### AW strategy

**Figure 2:**
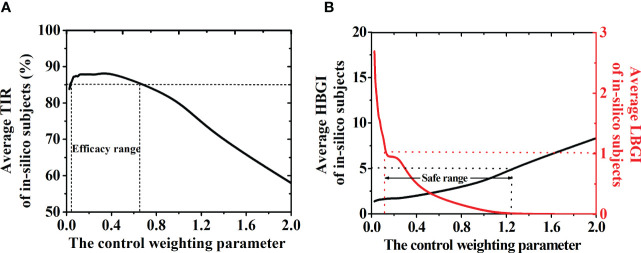
**(A)** TIR of the patient group with different control weighting parameter (*Λ*). The dotted lines represent the efficacy range of *Λ* (0.025 to 0.5), in which the TIR of the patient group is above 85%. **(B)** HBGI (black line) and LBGI (red line) values of the patient group with different *Λ*. The dotted lines represent the safe range of the control weighting parameter (0.125 to 1.25), in which the LBGI< 1.1 and HBGI<5.0. Thus, the selection range of the control weighting parameter should be 0.125 to 0.5.


*Λ* in Function (1) determines the weight of the control input deviation. Here, its effects on the GPC-based AP system were discussed and found that the average TIR of in-silico subjects decreased gradually when *Λ* was above 0.5 (TIR<85%, **Figure 2A**). In-silico patients had a high risk of hypoglycemia (LBGI>1.1, red line in **Figure 2B**) when *Λ* is smaller than 0.125, but a hyperglycemia phenomenon would also occur (HBGI>5.0, black line in **Figure 2B**) when *Λ* is above 1.25. Therefore, more insulin will be injected, and the risk of hypoglycemia increases when *Λ* has a low value. In this study, an AW strategy was proposed to ensure the efficacy and safety of the GPC-controller, in which *Λ* varied with the BG fluctuations. The dotted lines in **Figure 2A** represent the efficacy range of *Λ* (i.e., 0.03<*Λ<*0.5), in which the average TIR of the in-silico subjects is above 85%. The safe range of *Λ* (i.e., 0.125<*Λ<*1.25), in which the average LBGI value of the in-silico subjects is below 1.1 and their average HBGI average is below 5.0, is demonstrated in **Figure 2B**. Thus, the selection range of the control weighting parameter should be 0.125 to 0.5. The AW strategy is designed and shown in Function 4, where *θ* = 2 and $\bar{y\;}$ = 130 mg/dl. *y*(*k*) is the BG measured at *k* step. The upper and lower limits of *Λ* are defined as 0.125 and 0.5, respectively. In the AW strategy, the value of Λ would decrease when the BG value *y*(*k*) increases above the optimal BG value $\bar{y\;}$ . The insulin injection rate would fluctuate more violently to avoid hyperglycemia. Furthermore, Λ would increase gradually when the BG value y(k) reduces to the optimal BG value $\bar{y\;}$ . The insulin injection rate would be more stable in that progress.

(4) $$\lambda =\{\begin{array}{c} {\left[\theta \times \left(1+\frac{\left|y_{\left(k\right)}-\bar{y}\right|}{\bar{y}}\right)\right]}^{\left[y_{\left(k-1\right)}-y_{\left(k\right)}\right]}\;\;\;\;\;y_{\left(k-1\right)}-y_{\left(k\right)}<0\;\;\\ \;{\left[\theta \times \left(1+\frac{\left|y_{\left(k\right)}-\bar{y}\right|}{\bar{y}}\right)\right]}^{-1}\;\;\;\;\;\;\;\;\;\;\;\;y_{\left(k-1\right)}-y_{\left(k\right)}\geq 0 \end{array}$$

## Results

### In-silico tests of the GPC controller with strict conditions

First, the proposed GPC controller in our previous research was tested with nine in-silico subjects under strict conditions, including a pump with noise and error, a CGM sensor with noise and error, and a high CHO intake. Considering the effects of the CGM error and pump error, the scenario was repeated 30 times for each in-silico subject (Sections 2.1 and 2.2).

**Figure 3:**
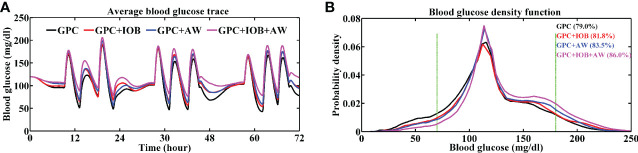
Average BG trace **(A)** and BG density **(B)** of 9 in-silico subjects. The TIR of the patient group with different controller is labeled in brackets. Green lines denote the euglycemic range. Black line represents the test with GPC controller, red line represents the test with GPC+IOB controller, blue line represents the test with GPC+AW controller and the pink line represents the test with GPC+IOB+AW controller.

**Figure 4:**
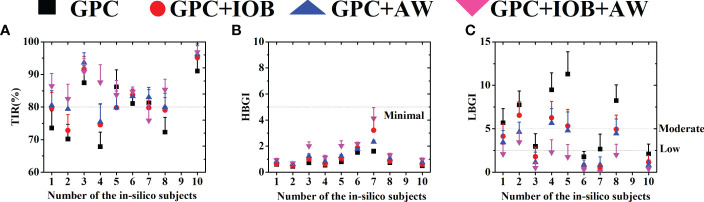
**(A)** The TIR of 9 in-silico subjects. **(B)** The HBGI of 9 in-silico subjects. **(C)** The LBGI of 9 in-silico subjects. Black squares represent the test with GPC controller, red circulars represent the test with GPC+IOB controller, blue triangles represent the test with GPC+AW controller and the pink triangles represent the test with GPC+IOB+AW controller.

The BG trace of each in-silico subject was recorded, and their average BG was 103.1 ± 10.7 mg/dL. The merged BG trace and density of nine subjects were shown in **Figures 3A, B** (black lines). The average TIR of the patient group was 79.0% ± 8.3% (average ± standard deviation). The TIR of each subject was shown in **Figure 4A** (black squares). Five of them (No. 03, 05, 06, 07, and 10) had large TIR above 80% (i.e., 87.5% ± 5.2%, 86.2% ± 5.2%, 81.1% ± 1.9%, 81.3% ± 4.7% and 91.1% ± 4.8%). The TIR values of three subjects (i.e., Nos. 01, 02, and 08) were between 70% and 80% (i.e., 73.6% ± 5.3%, 70.2% ± 4.5%, and 72.3% ± 4.5%, respectively). However, the TIR of subject No. 4 was only 67.9% ± 4.4%.

**Figure 5:**
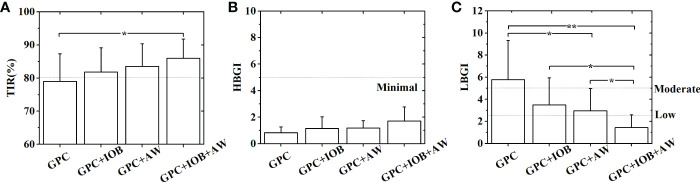
Statistical analyses of the TIR **(A)**, HBGI **(B)** and LBGI **(C)** of 9 in-silico subjects with different GPC controllers. Statistical analysis was performed in two steps: First, F-tests were performed to compare variances of the data sets. Then T-tests are conducted to further examine the differences between the data sets. Unequal variance T-tests were applied when the variances were not equal. *p< 0.05. **p< 0.01.

Percentages of time spent in the severe hypoglycemia range (BG ≤ 50 mg/dL), the hypoglycemia range (BG ≤ 70 mg/dL), the hyperglycemia range (BG > 180 mg/dL), and the severe hyperglycemia range (BG > 300 mg/dL) of each subject are listed in **Table 1**. The average percentages of time spent in the severe hypoglycemia range, the hypoglycemia range, the hyperglycemia range, and the severe hyperglycemia range of the patient group were 10.1% ± 7.1%, 19.7% ± 10.2%, 3.3% ± 3.4%, and 0.0% ± 0.0%, respectively. Two indexes provided by the T1DMS software, namely, HBGI and LBGI, were calculated for each subject to identify whether they are prone to hyperglycemia or hypoglycemia (black squares in **Figures 4B, C**). The HBGI and LBGI values of the patient group showed normal distributions (**Figures S1A**, **S2A**, respectively). The HBGI values of 9 subjects were 0.6 ± 0.1, 0.5 ± 0.1, 0.7 ± 0.1, 0.5 ± 0.1, 0.8 ± 0.1, 1.5 ± 0.1, 1.6 ± 0.1, 0.7 ± 0.1, and 0.1 ± 0.6, indicating that all subjects had a minimal risk (HBGI<5.0) for hyperglycemia (**Figures 4B**, **5B**). The LBGI values of these 9 subjects were 5.7 ± 1.6, 7.8 ± 1.6, 2.9 ± 1.5, 9.5 ± 1.9, 11.3 ± 2.6, 1.8 ± 0.6, 2.6 ± 1.7, 8.2 ± 1.8, and 2.1 ± 1.1, respectively. Two subjects (No. 06 and 10) had a low risk (LBGI<2.5) for hypoglycemia, whereas another two subjects (No. 03 and 07) had a moderate risk (2.5≤LBGI<5.0). The five remaining subjects (No. 01, 02, 04, 05, and 08) had a high risk for hypoglycemia (**Figure 4C**). Therefore, the GPC controller needs to be improved further to prevent hypoglycemia in patients with T1D.

**Table 1:** Percentages of time spent in the severe hypoglycemia (BG ≤ 50 mg/dL) range, the hypoglycemia (BG ≤ 70 mg/dL) range, the hyperglycemia range (BG > 180 mg/dL), and the severe hyperglycemia range (BG > 300 mg/dL) of each in-silico subject with the GPC controller.

Subject No.	Percentages of time spent in the severe hypoglycemia (BG ≤ 50 mg/dL) range	Percentages of time spent in the hypoglycemia (BG ≤ 70 mg/dL) range	Percentages of time spent in the hyperglycemia (BG ≥ 180 mg/dL) range	Percentages of time spent in the severe hyperglycemia (BG ≥ 300 mg/dL) range
01	10.7% ± 3.3%	24.2% ± 5.5%	2.3% ± 1.2%	0.0% ± 0.0%
02	16.0% ± 3.5%	28.9% ± 4.5%	0.9% ± 0.6%	0.0% ± 0.0%
03	4.3% ± 2.9%	11.9% ± 5.2%	0.6% ± 0.6%	0.0% ± 0.0%
04	17.6% ± 4.7%	30.6% ± 4.5%	1.5% ± 0.9%	0.0% ± 0.0%
05	17.9% ± 3.9%	29.2% ± 5.1%	2.6% ± 0.7%	0.0% ± 0.0%
06	3.1% ± 1.5%	8.7% ± 2.2%	10.2% ± 1.3%	0.0% ± 0.0%
07	3.9% ± 3.0%	10.4% ± 4.7%	8.3% ± 1.3%	0.0% ± 0.0%
08	14.8% ± 3.6%	24.9% ± 4.7%	2.8% ± 1.3%	0.0% ± 0.0%
10	2.5% ± 2.6%	8.4% ± 4.7%	0.6% ± 0.6%	0.0% ± 0.0%

### Tests of the GPC+IOB controller

Hypoglycemia occurs because too much insulin is injected into the body. Thus, an IOB calculator was introduced to the GPC controller to calculate the insulin that remains active within the body, which will be subtracted at each step (Section 2.3).

The GPC+IOB controller was tested with the same scenario in Section 3.1. The BG trace of each in-silico subject was recorded, and their average BG was 110.5 ± 14.5 mg/dL. The merged BG trace and density of the 9 subjects were shown in **Figures 3A, B** (red lines), respectively. The average TIR of the patient group was 81.8% ± 7.3%. The TIR of each subject were 79.6% ± 4.8%, 72.8% ± 4.9%, 91.6% ± 4.0%, 74.6% ± 6.5%, 79.8% ± 4.4%, 83.8% ± 1.8%, 79.8% ± 5.6%, 79.1% ± 3.9%, and 95.1% ± 3.7% (red circulars in **Figure 4A**). Compared with the test results in the GPC controller, the TIR of seven subjects (Nos. 01, 02, 03, 04, 06, 08, and 10) increased by 6.1%, 2.7%, 4.1%, 6.7%, 2.7%, 6.7%, and 4.1%, respectively. Meanwhile, the TIR of the two other subjects (No. 05 and 07) decreased by 6.4% and 1.5%, respectively. Although the TIR of the patient group with GPC+IOB controller was higher than that with the GPC controller, the statistical analysis (i.e., F-test and T-test) showed no significant differences (**Figure 5A**).

The percentages of time spent in the severe hypoglycemia range, the hypoglycemia range, the hyperglycemia range, and the severe hyperglycemia range of each subject was listed in **Table 2**. The average percentages of time spent in the severe hypoglycemia range, the hypoglycemia range, the hyperglycemia range, and the severe hyperglycemia range of the patient group were 5.9% ± 5.4%, 13.3% ± 9.7%, 4.9% ± 6.2%, and 0.0% ± 0.0%, respectively. Thus, the hypoglycemia risk of the patient group decreased compared with the results in Section 3.1. The HBGI and LBGI values of each subject with GPC+IOB controller were demonstrated in **Figures 4B, C** (red circulars), respectively. The HBGI values of the 9 subjects were 0.6 ± 0.1, 0.5 ± 0.1, 1.0 ± 0.2, 0.6 ± 0.1, 1.0 ± 0.1, 1.9 ± 0.1, 3.2 ± 1.0, 0.9 ± 0.1, and 0.6 ± 0.1, respectively. All subjects had minimal risk for hyperglycemia (**Figures 4B**, **5B**). The LBGI values of the 9 subjects were 4.1 ± 1.4, 6.6 ± 1.6, 1.8 ± 1.1, 6.3 ± 1.9, 5.3 ± 1.9, 0.7 ± 0.3, 0.5 ± 0.4, 4.9 ± 1.6, and 1.2 ± 0.8. Four of them (No. 03, 06, 07, and 10) had a low risk for hypoglycemia, two (No. 01 and 08) had a moderate risk, and the remaining three (No. 02, 04, and 05) have a high risk (**Figure 4C**). In relation to the test results of the GPC controller, the LBGI values of the 9 subjects decreased by 1.6%, 1.2%, 1.2%, 3.2%, 6.0%, 1.1%, 2.2%, 3.3%, and 1.0%. The HBGI and LBGI values of the patient group showed normal distributions (**Figures S1B**, **S2B**, respectively), thus F-test and T-test were performed to verify whether the GPC+IOB controller had a better performance. However, the statistical analysis showed that the improvement of the hypoglycemia was not significant (**Figure 5C**).

**Table 2:** Percentages of time spent in the severe hypoglycemia (BG ≤ 50 mg/dL) range, the hypoglycemia (BG ≤ 70 mg/dL) range, the hyperglycemia range (BG > 180 mg/dL), and the severe hyperglycemia range (BG > 300 mg/dL) of each in-silico subject with the GPC+IOB controller.

Subject No.	Percentages of time spent in the severe hypoglycemia (BG ≤ 50 mg/dL) range	Percentages of time spent in the hypoglycemia (BG ≤ 70 mg/dL) range	Percentages of time spent in the hyperglycemia (BG ≥ 180 mg/dL) range	Percentages of time spent in the severe hyperglycemia (BG ≥ 300 mg/dL) range
01	7.4% ± 2.7%	18.4% ± 5.0%	1.9% ± 1.0%	0.0% ± 0.0%
02	13.6% ± 3.6%	26.0% ± 4.8%	1.1% ± 0.7%	0.0% ± 0.0%
03	2.6% ± 2.5%	7.1% ± 4.0%	1.3% ± 0.8%	0.0% ± 0.0%
04	10.7% ± 4.1%	23.8% ± 6.6%	1.7% ± 0.9%	0.0% ± 0.0%
05	8.3% ± 3.2%	16.7% ± 4.9%	3.4% ± 1.0%	0.0% ± 0.0%
06	0.3% ± 0.7%	3.7% ± 1.9%	12.5% ± 0.9%	0.0% ± 0.0%
07	0.3% ± 0.8%	1.9% ± 2.1%	18.3% ± 5.7%	0.0% ± 0.0%
08	9.0% ± 3.4%	17.7% ± 4.2%	3.2% ± 1.3%	0.0% ± 0.0%
10	1.0% ± 1.3%	4.4% ± 3.7%	0.5% ± 0.6%	0.0% ± 0.0%

### Tests of the GPC+AW controller

Control weighting parameter, *Λ*, determines the weight of the control input deviation. A small *Λ* value means a fast change in the insulin injection rate, and a high *Λ* value means a stable insulin injection rate. Eren M. et al. also suggested that the *Λ* value played an important role in hypoglycemia prevention (15). In the present work, we discussed its effect on the efficacy and safety of the GPC-based AP. The efficacy of the GPC controller (i.e., the TIR of the patient group) greatly decreased to 60% as λ increased to 2 (**Figure 2A**). Although the hyperglycemia risk of the patients (i.e., HBGI value) decreased with λ (black line in **Figure 2B**), their hypoglycemia risk (i.e., LBGI value) rapidly increased (red line in **Figure 2B**). Here, an AW strategy was proposed, in which the λ value varied with the BG of the patient (Section 2.4). In short, λ would have a low value when the BG is increasing sharply but adopts a high value when the BG is decreasing gradually.

The GPC+AW controller was tested with T1DMS software following the same scenario as before. The BG trace of each in-silico subject was recorded, and their average BG was 112.9 ± 11.1 mg/dL. The merged BG trace and density of the 9 subjects were shown in **Figures 3A, B** (blue lines), respectively. The average TIR of the patient group was 83.5% ± 6.9%. The TIR of each subject were 80.5% ± 4.5%, 79.4% ± 3.6%, 93.6% ± 3.1%, 75.4% ± 5.4%, 79.9% ± 5.1%, 83.4% ± 1.9%, 83.0% ± 3.0%, 80.0% ± 4.2%, and 96.1% ± 3.2% (blue triangles in **Figure 4A**). In relation to the test results with the GPC controller, the TIR of eight subjects (No. 01, 02, 03, 04, 06, 07, 08, and 10) increased by 7.0%, 9.2%, 6.2%, 7.6%, 2.3%, 1.8%, 7.7%, and 5.1%, respectively. However, TIR of subject No. 05 decreased by 6.3%. Statistical analysis was further performed, but no significant difference was found (**Figure 5A**).

The percentages of time spent in the severe hypoglycemia range, the hypoglycemia range, the hyperglycemia range, and the severe hyperglycemia range of each subject were listed in **Table 3**. The average percentages of time spent in the severe hypoglycemia range, the hypoglycemia range, the hyperglycemia range, and the severe hyperglycemia range of the patient group were 4.9% ± 4.5%, 11.4% ± 8.2%, 5.1% ± 4.4%, and 0.0% ± 0.0%, respectively. The HBGI and LBGI values of the patient group were calculated and showed normal distributions, as shown in **Figures S1C**, **S2C**. **Figures 4B, C** (blue triangles) showed the HBGI and LBGI values of each subject with GPC+AW controller, respectively. The HBGI values of the 9 subjects were 0.8 ± 0.1, 0.6 ± 0.1, 1.2 ± 0.2, 0.8 ± 0.1, 1.2 ± 0.1, 1.8 ± 0.1, 2.3 ± 0.1, 1.0 ± 0.1, and 0.8 ± 0.1. All subjects had minimal risk for hyperglycemia (**Figures 4B**, **5B**). The LBGI values of the 9 subjects were 3.5 ± 1.3, 4.6 ± 1.1, 1.2 ± 1.2, 5.7 ± 1.7, 4.8 ± 2.1, 0.9 ± 0.5, 0.9 ± 0.9, 4.5 ± 1.7, and 0.8 ± 0.6, respectively. Four of them (No. 03, 07, 08, and 10) had a low risk for hypoglycemia, four (No. 01, 02, 05, and 08) had a moderate risk, and only one subject (No. 04) has a high risk (**Figure 4C**). In relation to the test results of the GPC controller, the LBGI values of the 9 subjects decreased by 2.2%, 3.1%, 1.8%, 3.8%, 6.5%, 0.9%, 1.8%, 3.8%, and 1.3%. Statistical analysis showed that the LBGI values of the patient group with GPC+AW controller was significantly lower than those with the GPC controller, indicating that the hypoglycemia was significantly improved (p<0.05) (**Figure 5C**).

**Table 3:** Percentages of time spent in the severe hypoglycemia (BG ≤ 50 mg/dL) range, the hypoglycemia (BG ≤ 70 mg/dL) range, the hyperglycemia range (BG > 180 mg/dL), and the severe hyperglycemia range (BG > 300 mg/dL) of each in-silico subject with the GPC+AW controller.

Subject No.	Percentages of time spent in the severe hypoglycemia (BG ≤ 50 mg/dL) range	Percentages of time spent in the hypoglycemia (BG ≤ 70 mg/dL) range	Percentages of time spent in the hyperglycemia (BG ≥ 180 mg/dL) range	Percentages of time spent in the severe hyperglycemia (BG ≥ 300 mg/dL) range
01	5.4% ± 3.0%	16.2% ± 4.5%	3.2% ± 1.3%	0.0% ± 0.0%
02	9.5% ± 2.7%	18.9% ± 3.7%	1.7% ± 0.8%	0.0% ± 0.0%
03	1.6% ± 2.2%	4.1% ± 3.2%	2.3% ± 1.2%	0.0% ± 0.0%
04	9.8% ± 3.7%	22.3% ± 5.4%	2.2% ± 1.1%	0.0% ± 0.0%
05	7.2% ± 3.5%	14.9% ± 5.1%	5.2% ± 0.9%	0.0% ± 0.0%
06	0.9% ± 1.4%	4.8% ± 2.1%	11.8% ± 1.0%	0.0% ± 0.0%
07	1.1% ± 1.7%	3.2% ± 3.2%	13.7% ± 1.3%	0.0% ± 0.0%
08	7.9% ± 3.1%	15.2% ± 4.2%	4.7% ± 1.2%	0.0% ± 0.0%
10	0.6% ± 1.1%	2.7% ± 3.3%	1.1% ± 0.6%	0.0% ± 0.0%

### Tests of the GPC+IOB+AW controller

Lastly, we tested the GPC+IOB+AW controller. The BG trace of each in-silico subject was recorded, and their average BG was 122.4 ± 14.2 mg/dL. The merged BG trace and density of the 9 subjects were shown in **Figures 3A, B** (pink lines), respectively. The average TIR of the patient group was 86.0% ± 5.8%. The TIR of each subject were 86.4% ± 3.9%, 82.5% ± 4.6%, 90.8% ± 4.0%, 87.6% ± 5.4%, 83.7% ± 4.5%, 84.7% ± 1.4%, 75.9% ± 4.9%, 85.3% ± 3.2%, and 96.9% ± 2.0%, respectively (pink triangles in **Figure 4A**). In relation to the test results with the GPC controller, the TIR of seven subjects (No. 01, 02, 03, 04, 06, 08, and 10) increased by 12.9%, 12.3%, 3.3%, 19.7%, 3.6%, 13.0%, and 5.8%, respectively, whereas TIR of two subjects (No. 05 and 07) decreased by 2.5% and 5.4%, respectively. Statistical analysis showed that the TIR of the patient group with the GPC+AW+IOB controller was significantly higher than that with the GPC controller (p<0.05). Thus, the efficacy of the GPC controller was significantly improved with the IOB and AW strategies (**Figure 5A**).

The percentages of time spent in the severe hypoglycemia range, the hypoglycemia range, the hyperglycemia range, and the severe hyperglycemia range of each subject were listed in **Table 4**. The average percentages of time spent in the severe hypoglycemia range, the hypoglycemia range, the hyperglycemia range, and the severe hyperglycemia range of the patient group were 2.1% ± 2.9%, 5.8% ± 6.0%, 8.2% ± 7.1%, and 0.0% ± 0.0%, respectively. Seemingly, the hypoglycemia risk declined further. The HBGI and LBGI values of the patient group were calculated. As shown in **Figures S1D**, **S2D**, the data showed normal distributions. The HBGI and LBGI values of each subject with GPC+IOB+AW controller were shown in **Figures 4B, C** (pink triangles), respectively. The HBGI values of the 9 subjects were 0.9 ± 0.1, 0.7 ± 0.1, 2.0 ± 0.3, 1.1 ± 0.2, 2.0 ± 0.4, 2.2 ± 0.1, 4.1 ± 0.8, 1.3 ± 0.2, and 1.0 ± 0.1, respectively. All subjects had minimal risk for hyperglycemia (**Figures 4B**, **5B**). The LBGI values of the 9 subjects were 2.1 ± 1.1, 3.5 ± 1.2, 0.5 ± 0.6, 2.3 ± 1.4, 1.8 ± 1.4, 0.3 ± 0.3, 0.1 ± 0.2, 2.0 ± 1.2, and 0.4 ± 0.3, respectively. Eight of them (No. 01, 03, 04, 05, 06, 07, 08, and 10) had a low risk for hypoglycemia, and only one subject (No. 02) had a moderate risk (**Figure 4C**). In relation to the test results of the GPC controller, the LBGI values of the 9 subjects decreased by 3.6%, 4.3%, 2.5%, 7.2%, 9.5%, 1.5%, 2.6%, 6.2%, and 1.7%, respectively. Statistical analysis (i.e., F-test and T-test) showed that the LBGI values of the patient group with GPC+IOB+AW controller were significantly lower than those with the GPC controller (p<0.01), GPC+IOB controller (p<0.05), and GPC+AW controller (p<0.05) (**Figure 5C**). Thus, the GPC+IOB+AW controller achieves optimal glycemic control and minimizes the risk of hypoglycemia in patients with T1D.

**Table 4:** Percentages of time spent in the severe hypoglycemia (BG ≤ 50 mg/dL) range, the hypoglycemia (BG ≤ 70 mg/dL) range, the hyperglycemia range (BG > 180 mg/dL), and the severe hyperglycemia range (BG > 300 mg/dL) of each in-silico subject with the GPC+IOB+AW controller.

Subject No.	Percentages of time spent in the severe hypoglycemia (BG ≤ 50 mg/dL) range	Percentages of time spent in the hypoglycemia (BG ≤ 70 mg/dL) range	Percentages of time spent in the hyperglycemia (BG ≥ 180 mg/dL) range	Percentages of time spent in the severe hyperglycemia (BG ≥ 300 mg/dL) range
01	2.7% ± 2.6%	9.4% ± 4.4%	4.1% ± 1.2%	0.0% ± 0.0%
02	6.6% ± 2.4%	15.3% ± 4.6%	2.2% ± 0.8%	0.0% ± 0.0%
03	0.7% ± 1.3%	2.1% ± 2.6%	7.1% ± 3.3%	0.0% ± 0.0%
04	2.8% ± 2.8%	9.2% ± 5.6%	3.2% ± 1.1%	0.0% ± 0.0%
05	2.6% ± 2.7%	5.9% ± 4.6%	10.4% ± 2.8%	0.0% ± 0.0%
06	0.1% ± 0.5%	1.3% ± 1.7%	14.0% ± 0.9%	0.0% ± 0.0%
07	0.0% ± 0.2%	0.2% ± 0.9%	23.8% ± 4.9%	0.0% ± 0.0%
08	3.7% ± 2.6%	8.1% ± 3.6%	6.6% ± 1.3%	0.0% ± 0.0%
10	0.1% ± 0.4%	1.1% ± 1.8%	2.0% ± 0.9%	0.0% ± 0.0%

### Evaluation of the GPC+IOB+AW controller with 100 in-silico subjects and normal CHO intakes

**Figure 6:**
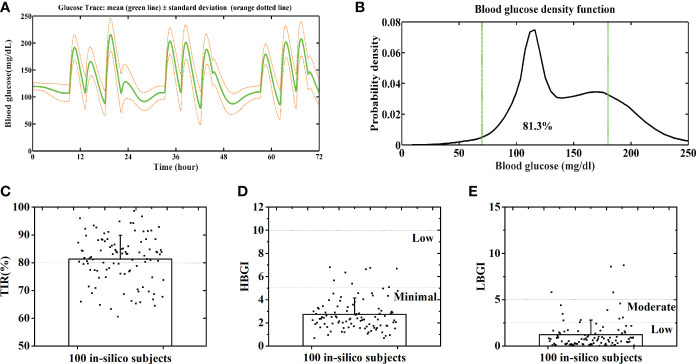
Average BG trace **(A)** and BG density **(B)** of 100 in-silico subjects. Distributions of the TIR **(C)**, HBGI **(D)** and LBGI **(E)** of 100 in-silico subjects. Green lines denote the euglycemic range.

The GPC+IOB+AW controller was further sent to the Epsilon Group and evaluated with 100 in-silico subjects. The DIA of the IOB calculator was set as 3 h without any personalized data. The same scenario and parameter settings in Section 3.1 were repeated 10 times for each in-silico subject. The merged BG trace and density of 100 subjects were shown in **Figures 6A, B**, respectively. The average TIR value of the patient group is 81.3% ± 8.6%, and TIR values of 85 subjects were above 70% (**Figure 6C**). The percentages of time spent in the severe hypoglycemia range, the hypoglycemia range, the hyperglycemia range, and the severe hyperglycemia range of each subject was listed in **Table S1**. The average percentages of the time spent in the severe hypoglycemia range, the hypoglycemia range, the hyperglycemia range, and the severe hyperglycemia range of the patient group were 1.7% ± 3.6%, 4.4% ± 6.2%, 14.7% ± 9.0%, and 0.1% ± 0.6%, respectively. All 100 in-silico subjects had a low risk for hyperglycemia, with the average HBGI value of 2.7 ± 1.4 (**Figure 6D**). A total of 87 subjects had a low risk, 9 subjects had a moderate risk, and 4 subjects had a high risk for hypoglycemia (**Figure 6E**). The average LGBI value of the patient group is 1.2 ± 1.6. Therefore, the GPC+IOB+AW controller realized effective and safe BG control for the majority of the population.

**Figure 7:**
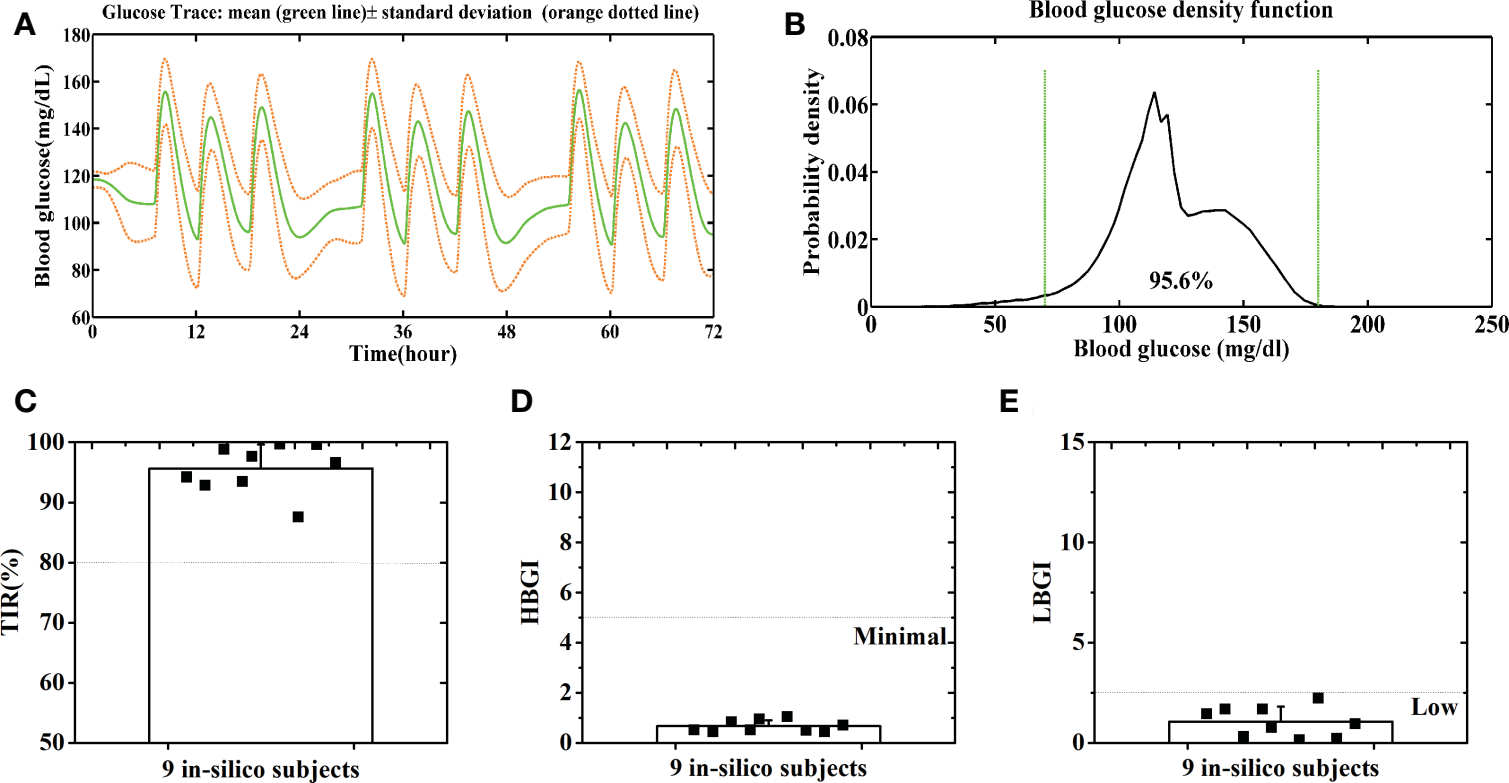
Average BG trace **(A)** and BG density **(B)** of 9 in-silico subjects with a normal CHO intake. Distributions of the TIR **(C)**, HBGI **(D)** and LBGI **(E)** of 9 in-silico subjects with a normal CHO intake. Green lines denote the euglycemic range.

People might be concerned that the GPC+IOB+AW is only applicable to those patients with high CHO intake. Hence, we also tested its performance with normal CHO intakes, as stated as follows: 30 g of CHO at 7 AM, 30 g at 12 PM, and 30 g at 6 PM daily, 3 days. **Figures 7A, B** show the merged BGC trace and density of the nine subjects, respectively. The average TIR of the patient group was 95.6% ± 3.9% (**Figure 7C**). The percentages of the time spent in the severe hypoglycemia range, the hypoglycemia range, the hyperglycemia range, and the severe hyperglycemia range of each subject were listed in **Table S2**. The average percentages of time spent in the severe hypoglycemia range, the hypoglycemia range, the hyperglycemia range, and the severe hyperglycemia range of the patient group were 1.4% ± 2.4%, 4.4% ± 5.2%, 0.0% ± 0.0%, and 0.0% ± 0.0%, respectively. All subjects had low risks for hyperglycemia and hypoglycemia, with the average HBGI and LBGI of 0.66 ± 0.23 and 1.05 ± 0.75, respectively (**Figures 7D, E**). Thus, the GPC+IOB+AW controller only focuses on the BGC variances and can handle different CHO intakes.

## Discussions

T1D occurs most frequently in children and young adults. In 2021, over 1.2 million children and adolescents had T1DM, and that number is increasing annually. Multiple daily injections, glucose monitoring, structured diabetes education, and expert medical care were great challenges for these young people and had affected their normal lives seriously. An automatic AP is a promising tool to solve that problem (2, 3).

The aim of our research is to develop an automated AP without any user interaction or personalized data. Thus, we proposed an intelligent controller based on GPC for AP, which only regards the BG levels provided by the CGM without meal announcements. It realized effective BG control in our previous research and was further tested here with more strict conditions. Although it is effective in hyperglycemia prevention, hypoglycemia risk for patients increased to a high level. Thus, the GPC controller needs further improvement.

**Figure 8:**
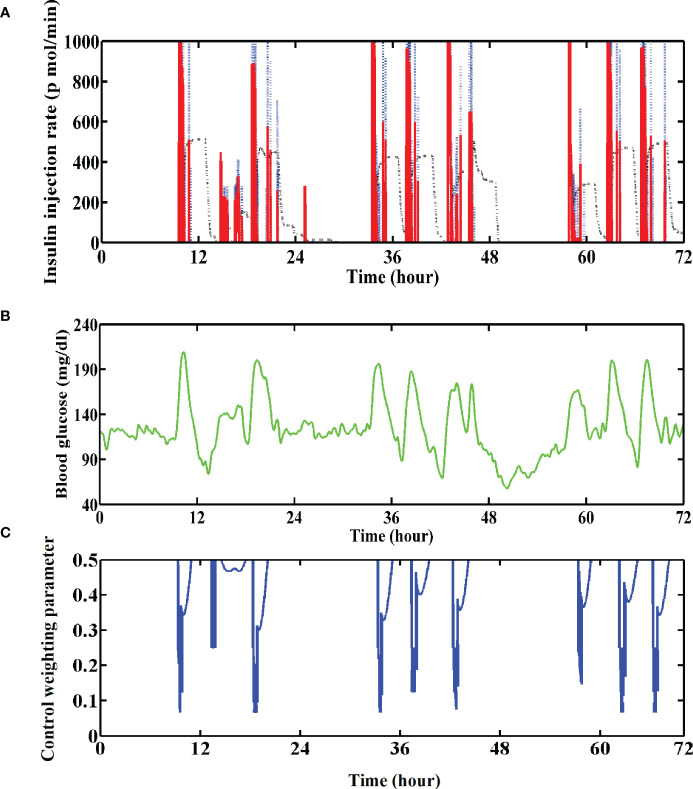
**(A)** Insulin injection rate for the No. 01 subject with the GPC+IOB+AW controller. The blue dotted line denotes the ideal insulin injection rate calculated by the GPC controller, the black dotted line denotes the estimated active insulin amount in the body and the red line denotes their subtraction. **(B)** Variations of the BG trace of the No. 01 subject with GPC+IOB+AW controller. **(C)** Variations of the control weighting parameter of the No. 01 subject.

The IOB calculator, which could estimate the insulin amount that remains active within the patient’s body, was introduced into the GPC controller. For instance, the estimated active insulin amount for subject No. 01 is shown in **Figure 8A** (black dotted line), which was subtracted from the ideal insulin injection rate calculated by the GPC algorithm (blue dotted line in **Figure 8A**) at each step (Section 2.3). Thus, less insulin would be injected into the patient’s body (red line in **Figure 8A**). The test results with T1DMS showed that the hypoglycemia risk for each in-silico subject reduced with the GPC+IOB controller. However, we found that the IOB calculator needs a personalized data, DIA. The incorrect estimation of the DIA induces mismatch in the IOB and insulin injection, thereby resulting in hypoglycemia or hyperglycemia. Hence, determining the individualized DIA still remains a critical point nowadays (29).

To overcome the aforementioned problem, we proposed an AW strategy without requiring any individualized data. As shown in **Figures 8B, C**, the value of *Λ* sharply decreases when the BG is greatly increasing but adopts a high value when the BG is gradually decreasing. Thus, a large dose of insulin will be injected when the BG is increasing, but a smaller dose of insulin will be injected when the BG is decreasing. The test results with T1DMS showed that the hypoglycemia of the patient group was significantly improved with the GPC+AW controller, which is more effective than the GPC+IOB controller (**Figure 5C**). Therefore, the AW strategy has high usability and performance.

The efficacy of our GPC+IOB+AW controller is comparable with other controllers, such as the proportional integral derivative with double phase lead (PIDD) controller, the proportional-integral-derivative (PID) controller, the model predictive controller (MPC), the extended model predictive controller (EMPC), and the conventional proportional-derivative (PD) controller with the fuzzy P part (Fuzzy P+D). The TIR of the patient group with the aforementioned controllers were 77%, 72.6%, 79.6%, 84.3%, and 83%, respectively (23, 32, 33). However, its performance still needs improvement compared with current standard-of-care, such as the basal-bolus therapy. The TIR, HBGI, and LBGI of the in-silico subjects was 92%, 0.49, and 0.78 using the basal/bolus controller proposed by Fraser Cameron with the similar scenario, which is better than the results here, as well as the results obtained by other controllers (23). However, the basal-bolus therapy design requires user interaction and personalized data, including meal information, the carbohydrate-to-insulin ratio, and the correction factor. Moreover, the values of these factor may vary during the day (34). The most outstanding feature of our GPC+IOB+AW is that it realizes an automatic control and does not require user interaction, personalized data, and meal announcements.

Moreover, subsequent tests with real patients are still substantially needed because an in-silico population with an average TIR of 86% is only representative to a very limited proportion of real patients. Other modules, such as the carbohydrate on board, should also be considered in future research.

## Data availability statement

The original contributions presented in the study are included in the article/**Supplementary Material**. Further inquiries can be directed to the corresponding authors.

## Author contributions

HJ and WL conceived and designed the experiments. Modeling and simulation were conducted by WL, BL and TC. WL and YW analyzed the results and wrote the manuscript. All authors contributed to the article and approved the submitted version.
