# Supplementary material for: In-silico evaluation of an artificial pancreas achieving automatic glycemic control in patients with type 1 diabetes
Source: Front Endocrinol (Lausanne). 2023 Jan 30;14:1115436. doi: 10.3389/fendo.2023.1115436 (PMC9922739; doi:10.3389/fendo.2023.1115436)
Supplement: Supplementary file 1 [file DataSheet_1.pdf]

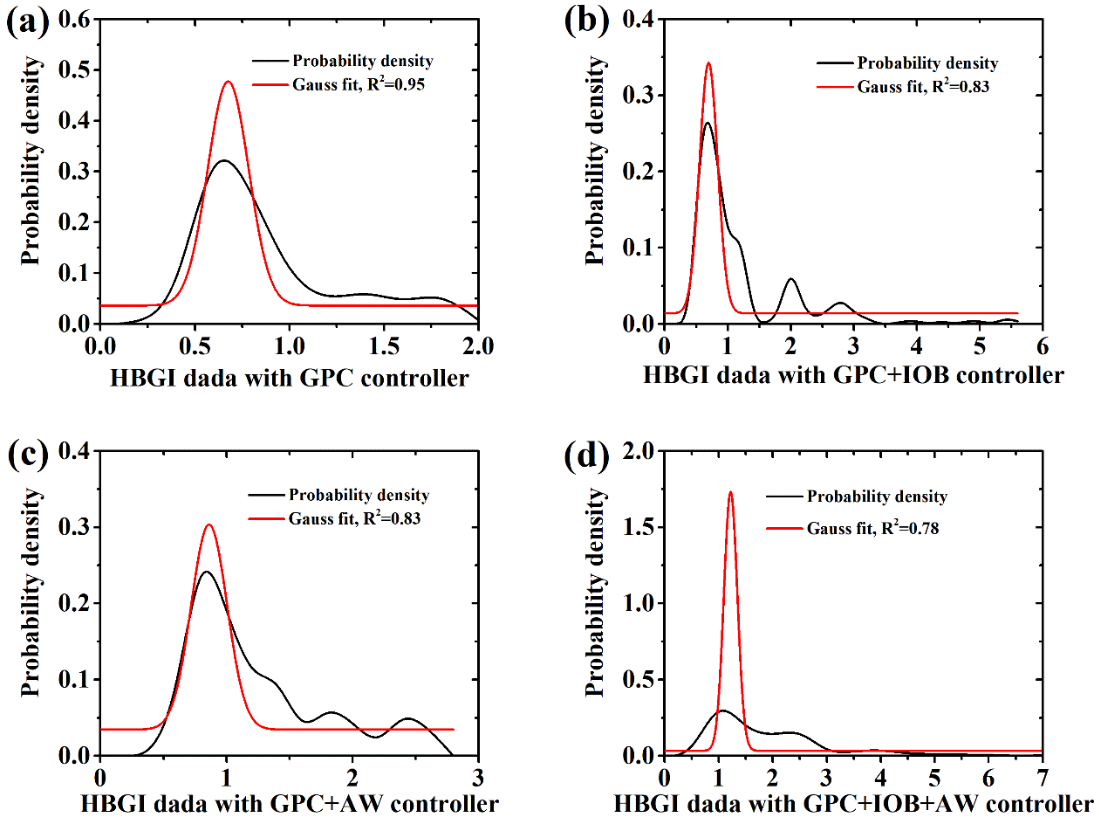

Figure S1 Distributions of the HBGI values of the in-silico patients with different GPC controllers. Black lines denote the probability and the red lines denote the gauss fit.

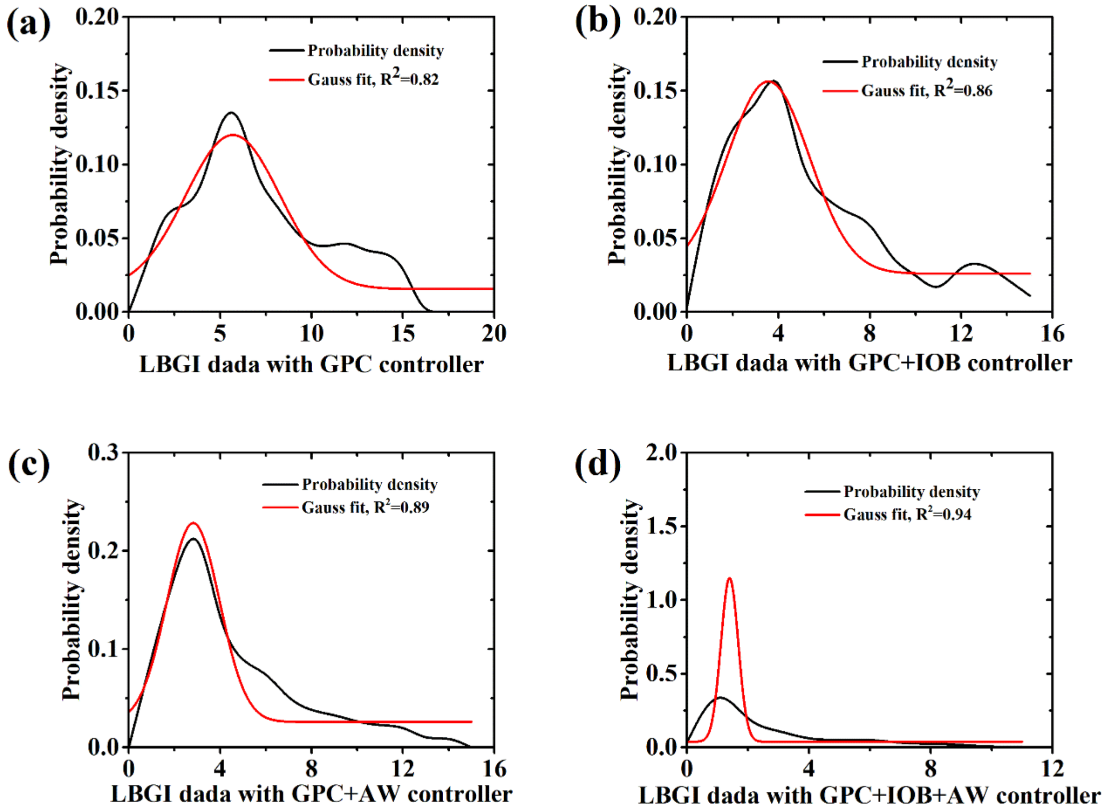

Figure S2 Distributions of the LBGI values of the in-silico patients with different GPC controllers. Black lines denote the probability and the red lines denote the gauss fit.
