# Supplementary material for: In-silico evaluation of an artificial pancreas achieving automatic glycemic control in patients with type 1 diabetes
Source: Front Endocrinol (Lausanne). 2023 Jan 30;14:1115436. doi: 10.3389/fendo.2023.1115436 (PMC9922739; doi:10.3389/fendo.2023.1115436)
Supplement: Supplementary file 2 [file DataSheet_2.pdf]

Table S1. Percentages of time spent in the severe hypoglycemia (BG ≤ 50 mg/dL) range, the hypoglycemia (BG ≤ 70 mg/dL) range, the hyperglycemia range (BG > 180 mg/dL), and the severe hyperglycemia range (BG > 300 mg/dL) of 100 in-silico subjects with the GPC+IOB+AW controller.

Subject No.	Percentages of time spent in the severe hypoglycemia (BG ≤ 50 mg/dL) range	Percentages of time spent in the hypoglycemia (BG ≤ 70 mg/dL) range	Percentages of time spent in the hyperglycemia (BG ≥ 180 mg/dL) range	Percentages of time spent in the severe hyperglycemia (BG ≥ 300 mg/dL) range
01	0.6 % ± 1.0 %	3.7 % ± 4.0 %	9.0 % ± 2.5 %	0.0 % ± 0.0 %
02	0.4 % ± 0.6 %	2.1 % ± 1.6 %	17.4 % ± 1.3 %	0.0 % ± 0.0 %
03	0.5 % ± 1.5 %	2.8 % ± 3.8 %	1.2 % ± 1.0 %	0.0 % ± 0.0 %
04	9.6 % ± 4.1 %	23.1 % ± 5.1 %	10.9 % ± 2.0 %	0.0 % ± 0.0 %
05	0.0 % ± 0.0 %	0.0 % ± 0.0 %	15.6 % ± 0.8 %	0.0 % ± 0.0 %
06	2.5 % ± 2.4 %	5.1 % ± 2.9 %	10.6 % ± 1.9 %	0.0 % ± 0.0 %
07	0.0 % ± 0.1 %	0.7 % ± 1.5 %	19.8 % ± 1.4 %	0.0 % ± 0.0 %
08	0.0 % ± 0.0 %	0.1 % ± 0.3 %	18.1 % ± 1.0 %	0.0 % ± 0.0 %
09	0.0 % ± 0.0 %	0.0 % ± 0.0 %	7.7 % ± 2.1 %	0.0 % ± 0.0 %
10	0.2 % ± 0.7 %	3.8 % ± 3.0 %	6.4 % ± 1.5 %	0.0 % ± 0.0 %
11	1.7 % ± 1.9 %	4.5 % ± 3 %	14.9 % ± 1.0 %	0.0 % ± 0.0 %
12	1.0 % ± 1.4 %	3.7 % ± 2.8 %	16.5 % ± 0.8 %	0.0 % ± 0.0 %
13	1.4 % ± 1.1 %	7.7 % ± 2 %	20.9 % ± 0.6 %	0.0 % ± 0.0 %
14	2.1 % ± 2.1 %	5.5 % ± 4.4 %	12.8 % ± 3.2 %	0.0 % ± 0.0 %
15	7.1 % ± 4.0 %	14.5 % ± 6.5 %	8.1 % ± 2.3 %	0.0 % ± 0.0 %
16	0.5 % ± 1.1 %	2.1 % ± 2.1 %	15.9 % ± 2.1 %	0.0 % ± 0.0 %
17	6.0 % ± 2.6 %	14.3 % ± 3.8 %	16.6 % ± 1.0 %	0.0 % ± 0.0 %
18	4.1 % ± 3.3 %	10.1 % ± 4.7 %	8.7 % ± 1.9 %	0.0 % ± 0.0 %
19	2.0 % ± 1.9 %	4.8 % ± 1.9 %	1.8 % ± 0.8 %	0.0 % ± 0.0 %
20	0.0 % ± 0.0 %	0.6 % ± 1.2 %	22.1 % ± 2.3 %	0.0 % ± 0.0 %
21	0.0 % ± 0.1 %	0.4 % ± 0.5 %	28.7 % ± 0.6 %	5.7 % ± 0.4 %
22	0.1 % ± 0.4 %	0.7 % ± 1.8 %	6.6 % ± 1.0 %	0.0 % ± 0.0 %
23	2.8 % ± 2.7 %	7.0 % ± 3.5 %	15.9 % ± 2.0 %	0.0 % ± 0.0 %
24	0.2 % ± 0.7 %	2.3 % ± 2.1 %	9.6 % ± 1.4 %	0.0 % ± 0.0 %
25	0.0 % ± 0.0 %	0.2 % ± 0.6 %	25.2 % ± 1.5 %	0.0 % ± 0.0 %
26	0.0 % ± 0.0 %	0.0 % ± 0.0 %	35.2 % ± 2.9 %	0.0 % ± 0.0 %
27	0.0 % ± 0.0 %	0.7 % ± 1.6 %	14.8 % ± 2.1 %	0.0 % ± 0.0 %
28	0.0 % ± 0.0 %	0.1 % ± 0.2 %	11.4 % ± 1.9 %	0.0 % ± 0.0 %
29	0.0 % ± 0.0 %	0.0 % ± 0.0 %	11.7 % ± 1.6 %	0.0 % ± 0.0 %
30	0.0 % ± 0.0 %	0.0 % ± 0.0 %	20.0 % ± 1.7 %	0.0 % ± 0.0 %
31	0.3 % ± 0.8 %	1.3 % ± 2.1 %	8.1 % ± 1.8 %	0.0 % ± 0.0 %
32	0.2 % ± 0.5 %	0.6 % ± 0.8 %	11.6 % ± 2.3 %	0.0 % ± 0.0 %
33	0.2 % ± 0.6 %	0.6 % ± 1.9 %	24.6 % ± 2.0 %	0.0 % ± 0.0 %
34	0.0 % ± 0.0 %	0.0 % ± 0.0 %	22.5 % ± 0.9 %	0.0 % ± 0.0 %
35	2.3 % ± 1.3 %	6.7 % ± 3.3 %	4.8 % ± 1.7 %	0.0 % ± 0.0 %
36	0.2 % ± 0.6 %	1.8 % ± 2.1 %	7.1 % ± 2.0 %	0.0 % ± 0.0 %

37	4.4 % ± 0.9 %	12.3 % ± 2.6 %	9.7 % ± 0.8 %	0.0 % ± 0.0 %
38	4.8 % ± 2.2 %	9.9 % ± 4.0 %	4.5 % ± 1.3 %	0.0 % ± 0.0 %
39	0.2 % ± 0.7 %	1.6 % ± 2.6 %	35.1 % ± 2.3 %	0.0 % ± 0.0 %
40	0.0 % ± 0.0 %	0.7 % ± 0.9 %	13.1 % ± 1.2 %	0.0 % ± 0.0 %
41	0.0 % ± 0.0 %	0.3 % ± 0.9 %	16.6 % ± 0.9 %	0.0 % ± 0.0 %
42	0.9 % ± 2.0 %	1.7 % ± 2.9 %	18.5 % ± 2.4 %	0.0 % ± 0.0 %
43	1.0 % ± 1.0 %	5.1 % ± 2.8 %	8.4 % ± 1.8 %	0.0 % ± 0.0 %
44	0.8 % ± 1.4 %	3.3 % ± 3.1 %	12.0 % ± 2.3 %	0.0 % ± 0.0 %
45	0.0 % ± 0.1 %	2.4 % ± 2.5 %	13.5 % ± 1.6 %	0.0 % ± 0.0 %
46	1.1 % ± 1.7 %	4.4 % ± 2.3 %	11.9 % ± 1.0 %	0.0 % ± 0.0 %
47	0.0 % ± 0.0 %	5.3 % ± 3.9 %	34.1 % ± 4.3 %	0.0 % ± 0.0 %
48	0.0 % ± 0.0 %	0.0 % ± 0.0 %	20.3 % ± 3.0 %	0.0 % ± 0.0 %
49	0.0 % ± 0.0 %	0.4 % ± 1.1 %	4.6 % ± 1.5 %	0.0 % ± 0.0 %
50	0.1 % ± 0.3 %	0.4 % ± 0.7 %	23.5 % ± 1.8 %	0.0 % ± 0.0 %
51	0.0 % ± 0.0 %	0.0 % ± 0.1 %	15.0 % ± 1.5 %	0.0 % ± 0.0 %
52	0.2 % ± 0.7 %	2.3 % ± 2.7 %	14.4 % ± 1.4 %	0.0 % ± 0.0 %
53	0.1 % ± 0.3 %	0.9 % ± 1.2 %	14.0 % ± 1.4 %	0.0 % ± 0.0 %
54	1.3 % ± 2.3 %	2.6 % ± 3.5 %	28.7 % ± 3.2 %	0.0 % ± 0.0 %
55	1.2 % ± 1.5 %	6.2 % ± 5.9 %	17.9 % ± 0.9 %	0.0 % ± 0.0 %
56	3.2 % ± 3.2 %	8.9 % ± 4.4 %	7.2 % ± 1.3 %	0.0 % ± 0.0 %
57	0.4 % ± 1.2 %	2.2 % ± 1.6 %	14.5 % ± 1.4 %	0.0 % ± 0.0 %
58	0.5 % ± 1.2 %	3.0 % ± 2.5 %	13.8 % ± 0.9 %	0.0 % ± 0.0 %
59	0.9 % ± 1.7 %	3.5 % ± 3.6 %	10.5 % ± 2.0 %	0.0 % ± 0.0 %
60	0.0 % ± 0.0 %	0.0 % ± 0.0 %	23.1 % ± 1.2 %	0.0 % ± 0.0 %
61	2.1 % ± 1.6 %	8.3 % ± 2.1 %	14.5 % ± 0.8 %	0.0 % ± 0.0 %
62	0.0 % ± 0.0 %	0.5 % ± 0.9 %	5.2 % ± 1.1 %	0.0 % ± 0.0 %
63	0.8 % ± 1.7 %	3.5 % ± 2.8 %	40.2 % ± 1.8 %	0.0 % ± 0.0 %
64	3.4 % ± 2.4 %	9.3 % ± 4.4 %	9.6 % ± 0.9 %	0.0 % ± 0.0 %
65	0.6 % ± 0.7 %	3.6 % ± 2.9 %	2.1 % ± 1.0 %	0.0 % ± 0.0 %
66	0.0 % ± 0.0 %	0.0 % ± 0.0 %	1.3 % ± 0.8 %	0.0 % ± 0.0 %
67	0.2 % ± 0.6 %	0.8 % ± 1.7 %	7.6 % ± 1.2 %	0.0 % ± 0.0 %
68	0.1 % ± 0.2 %	0.1 % ± 0.4 %	34.5 % ± 1.8 %	0.3 % ± 0.3 %
69	5.9 % ± 1.9 %	14.2 % ± 5.1 %	7.0 % ± 2.0 %	0.0 % ± 0.0 %
70	0.6 % ± 2.0 %	1.4 % ± 3.2 %	28.2 % ± 2.3 %	0.0 % ± 0.0 %
71	0.3 % ± 1.1 %	1.0 % ± 2.0 %	29.3 % ± 1.4 %	0.0 % ± 0.0 %
72	0.7 % ± 1.1 %	4.2 % ± 2.9 %	12.0 % ± 1.2 %	0.0 % ± 0.0 %
73	16.6 % ± 4.1 %	25.5 % ± 3.0 %	8.8 % ± 1.3 %	0.0 % ± 0.0 %
74	0.0 % ± 0.0 %	0.0 % ± 0.0 %	12.4 % ± 1.0 %	0.0 % ± 0.0 %
75	0.0 % ± 0.1 %	0.7 % ± 1.6 %	2.6 % ± 1.7 %	0.0 % ± 0.0 %
76	11.2 % ± 2.3 %	17.9 % ± 2.4 %	10.8 % ± 1.3 %	0.0 % ± 0.0 %
77	0.0 % ± 0.0 %	0.0 % ± 0.0 %	8.7 % ± 1.1 %	0.0 % ± 0.0 %
78	0.0 % ± 0.0 %	0.2 % ± 0.7 %	17.3 % ± 1.7 %	0.0 % ± 0.0 %
79	0.4 % ± 0.6 %	3.0 % ± 2.1 %	16.7 % ± 1.1 %	0.0 % ± 0.0 %
80	1.1 % ± 1.6 %	3.2 % ± 3.3 %	8.3 % ± 1.6 %	0.0 % ± 0.0 %

81	0.2 % ± 0.3 %	0.7 % ± 0.6 %	17.9 % ± 1.1 %	0.0 % ± 0.0 %
82	2.2 % ± 1.7 %	7.2 % ± 2.9 %	1.4 % ± 1.3 %	0.0 % ± 0.0 %
83	3.8 % ± 3.6 %	13.0 % ± 2.7 %	37.4 % ± 3.5 %	0.0 % ± 0.0 %
84	6.8 % ± 2.7 %	14.1 % ± 3.9 %	1.0 % ± 0.6 %	0.0 % ± 0.0 %
85	0.3 % ± 0.7 %	2.1 % ± 2.0 %	16.9 % ± 1.1 %	0.0 % ± 0.0 %
86	1.9 % ± 1.4 %	6.0 % ± 2.7 %	2.4 % ± 0.9 %	0.0 % ± 0.0 %
87	6.0 % ± 1.8 %	13.3 % ± 2.8 %	11.9 % ± 0.6 %	0.0 % ± 0.0 %
88	21.5 % ± 2.1 %	31 % ± 2.5 %	12.2 % ± 0.6 %	0.0 % ± 0.0 %
89	2.3 % ± 2.2 %	5.8 % ± 3.9 %	25.5 % ± 1.4 %	0.0 % ± 0.0 %
90	1.8 % ± 1.3 %	8.0 % ± 2.6 %	27.5 % ± 0.7 %	0.0 % ± 0.0 %
91	3.0 % ± 2.7 %	9.1 % ± 5.5 %	2.3 % ± 0.9 %	0.0 % ± 0.0 %
92	0.0 % ± 0.0 %	0.9 % ± 1.8 %	15.1 % ± 1.2 %	0.0 % ± 0.0 %
93	0.4 % ± 1.1 %	0.9 % ± 2.0 %	15.4 % ± 2.4 %	0.0 % ± 0.0 %
94	0.5 % ± 1.6 %	2.4 % ± 2.3 %	16.9 % ± 1.1 %	0.0 % ± 0.0 %
95	0.5 % ± 1.7 %	1.4 % ± 2.7 %	5.7 % ± 2.2 %	0.0 % ± 0.0 %
96	0.8 % ± 1.2 %	3.4 % ± 3.5 %	13.6 % ± 0.9 %	0.0 % ± 0.0 %
97	3.2 % ± 1.6 %	8.6 % ± 3.8 %	6.8 % ± 1.7 %	0.0 % ± 0.0 %
98	1.2 % ± 2.6 %	3.2 % ± 4.1 %	12.9 % ± 1.6 %	0.0 % ± 0.0 %
99	0.7 % ± 1.4 %	2.9 % ± 2.2 %	29.4 % ± 1.2 %	3.8 % ± 0.3 %
100	0.0 % ± 0.0 %	0.1 % ± 0.3 %	26.1 % ± 3.6 %	0.0 % ± 0.0 %

Table S2. Percentages of time spent in the severe hypoglycemia (BG ≤ 50 mg/dL) range, the hypoglycemia (BG ≤ 70 mg/dL) range, the hyperglycemia range (BG > 180 mg/dL), and the severe hyperglycemia range (BG > 300 mg/dL) of each in-silico subject with normal CHO intakes.

**Subject No.**	**Percentages of time spent in the severe hypoglycemia (BG ≤ 50 mg/dL) range**	**Percentages of time spent in the hypoglycemia (BG ≤ 70 mg/dL) range**	**Percentages of time spent in the hyperglycemia (BG ≥ 180 mg/dL) range**	**Percentages of time spent in the severe hyperglycemia (BG ≥ 300 mg/dL) range**
01	1.7 % ± 2.0 %	5.7 % ± 4.7 %	0.0 % ± 0.0 %	0.0 % ± 0.0 %
02	1.8 % ± 2.0 %	7.1 % ± 3.8 %	0.0 % ± 0.0 %	0.0 % ± 0.0 %
03	0.1 % ± 0.2 %	1.2 % ± 2.0 %	0.0 % ± 0.0 %	0.0 % ± 0.0 %
04	2.2 % ± 2.9 %	6.5 % ± 5.6 %	0.0 % ± 0.0 %	0.0 % ± 0.0 %
05	0.8 % ± 1.7 %	2.3 % ± 2.6 %	0.0 % ± 0.0 %	0.0 % ± 0.0 %
06	0.0 % ± 0.0 %	0.3 % ± 0.8 %	0.0 % ± 0.0 %	0.0 % ± 0.0 %
07	4.7 % ± 3.6 %	12.4 % ± 5.6 %	0.0 % ± 0.0 %	0.0 % ± 0.0 %
08	0.0 % ± 0.0 %	0.4 % ± 0.7 %	0.0 % ± 0.0 %	0.0 % ± 0.0 %
10	0.9 % ± 1.4 %	3.3 % ± 2.4 %	0.0 % ± 0.0 %	0.0 % ± 0.0 %
