# Supplementary material for: In-silico evaluation of an artificial pancreas achieving automatic glycemic control in patients with type 1 diabetes
Source: Front Endocrinol (Lausanne). 2023 Jan 30;14:1115436. doi: 10.3389/fendo.2023.1115436 (PMC9922739; doi:10.3389/fendo.2023.1115436)
Supplement: Supplementary file 3 [file DataSheet_3.pdf]

Appendix S1.

The standard deviation of the sensor error is hard to be estimated in real life and the Kalman Filter is just used to smooth the BG data slightly. The number of filters is 7. The initial condition for the estimated state is set as B_0. The initial condition for the estimated error covariance is set as B_1. The state transition matrix is set as B_2. The process noise covariance is set as B_3. The measurement matrix is set as B_4, and the measurement noise covariance is set as B_5. B_0~B_5 are shown as follows.

$$B_0 = \begin{bmatrix} 0 \\ 0 \\ 0 \\ 0 \\ 0 \\ 0 \end{bmatrix},\ B_1 = \begin{bmatrix} 10 & 0 & 0 & 0 & 0 & 0 \\ 0 & 10 & 0 & 0 & 0 & 0 \\ 0 & 0 & 10 & 0 & 0 & 0 \\ 0 & 0 & 0 & 10 & 0 & 0 \\ 0 & 0 & 0 & 0 & 10 & 0 \\ 0 & 0 & 0 & 0 & 0 & 10 \end{bmatrix},\ B_2 = \begin{bmatrix} 1 & 0 & 1 & 0 & 0 & 0 \\ 0 & 1 & 0 & 1 & 0 & 0 \\ 0 & 0 & 1 & 0 & 0 & 0 \\ 0 & 0 & 0 & 1 & 0 & 0 \\ 0 & 0 & 0 & 0 & 1 & 0 \\ 0 & 0 & 0 & 0 & 0 & 1 \end{bmatrix},$$

$$B_3 = \begin{bmatrix} 0.05 & 0 & 1 & 0 & 0 & 0 \\ 0 & 0.05 & 0 & 1 & 0 & 0 \\ 0 & 0 & 0.05 & 0 & 0 & 0 \\ 0 & 0 & 0 & 0.05 & 0 & 0 \\ 0 & 0 & 0 & 0 & 0.05 & 0 \\ 0 & 0 & 0 & 0 & 0 & 0.05 \end{bmatrix},\ B_4 = \begin{bmatrix} 1 & 0 & 0 & 0 & 0 & 0 \\ 0 & 1 & 0 & 0 & 0 & 0 \\ 0 & 0 & 0 & 0 & 1 & 0 \\ 0 & 0 & 0 & 0 & 0 & 1 \end{bmatrix},$$

$$B_5 = \begin{bmatrix} 1 & 0 & 0 & 0 \\ 0 & 1 & 0 & 0 \\ 0 & 0 & 1 & 0 \\ 0 & 0 & 0 & 1 \end{bmatrix}$$

Appendix S2.

The noise of the sensor (ε_n) is modelled by using an autoregressive moving average progress, as shown in the following functions.

$$\begin{cases} e_1 = v_1 \\ e_n = 0.7(e_{n-1} + v_n) \end{cases}$$

$$v_n \sim \varphi(0,1) (\text{Independent identically distributed})$$

$$\varepsilon_n = \zeta + \Lambda \sinh\left(\frac{e_n - \gamma}{\delta}\right)$$

e_n corresponds to the normally distributed time series. Parameters ζ, Λ, γ, and δ are the Johnson system (unbounded system) parameters that correspond to the empirical noise distributions. Here, $\zeta = -5.47$, Λ =15.9574, $\gamma = -0.5444$, and $\delta = 1.6898$.

The mean sensor BG reading ($\bar{s}$) and the mean error ($\bar{\varepsilon}$) for each reference BG were computed using the following function.

$$\bar{s} = \int_{s=0}^{600} s \times D(s, r) ds$$

$$\bar{\varepsilon} = \bar{s} - r$$

$D(s, r)$ is the distribution of the sensor readings for different glucose references by using kernel density estimation. s is the sensor reading, and r is the reference measure.

The partial autocorrelation function (PACF) is used to determine the sensor error in the T1DMS. PACF can be described as the correlation among errors at time t and $t + h$, $h = nT$, excluding the information transmitted through $t + T$, $t + 2T$, $t + 3T, \ldots, t + (n-1)T$. n is an integer, and T is set as 15 min. The larger the PACF is, the larger the error is. Here, the PACF is set as 0.7.

Pump noise and error are generated with two Gaussian-distributed random signal generators in the T1DMS, respectively. The pump noise is added to the basal delivery rate with a mean of 0 p mol/min and a standard deviation of 10 p mol/min. The pump error is added to the bolus rate calculated by the GPC controller. It has a mean of 0 p mol/min and a standard deviation of 50 p mol/min.
